# Appropriate incorporation of susceptibility-weighted magnetic resonance imaging into routine imaging protocols for accurate diagnosis of traumatic brain injuries: a systematic review

**DOI:** 10.25122/jml-2023-0487

**Published:** 2024-03

**Authors:** Osama Jaafari, Suliman Salih, Ajnas Alkatheeri, Muhamed Alshehri, Majedh Al-Shammari, Mousa Maeni, Abdullah Alqahtani, Wijdan Alomaim, Mohamed Hasaneen

**Affiliations:** 1 Radiology Department, Royal Commission Medical Center, King Fahad, Al-Nakheel, Yanbu, Saudi Arabia; 2 Department of Radiography and Medical Imaging, Fatima College of Health Sciences, Al Ain, United Arab Emirates; 3 Department of Radiology and Medical Imaging, Prince Sultan Military Medical City, Riyadh, Saudi Arabia; 4 Department of Radiological Sciences, College of Applied Medical Sciences, Najran University, Najran, Saudi Arabia; 5 Radiology and Medical Imaging Department, College of Applied Medical Sciences, Prince Sattam Bin Abdulaziz University, Alkharj, Saudi Arabia

**Keywords:** traumatic brain injury, magnetic resonance imaging, susceptibility-weighted imaging, traumatic axonal injury

## Abstract

Traumatic brain injury (TBI) results from physical or traumatic injuries to the brain’s surrounding bony structures and associated tissues, which can lead to various sequelae, including simple concussion, acute epidural hematoma, parenchymal contusions, subarachnoid hemorrhage, diffuse axonal injury, and chronic traumatic encephalopathy. Susceptibility-weighted imaging (SWI) has enhanced the accuracy of neuroimaging for these injuries. SWI is based on 3D gradient echo magnetic resonance imaging (MRI) with long echo times and flow compensation. Owing to its sensitivity to deoxyhemoglobin, hemosiderin, iron, and calcium, SWI is extremely informative and superior to conventional MRI for the diagnosis and follow-up of patients with acute, subacute, and prolonged hemorrhage. This systematic review aimed to evaluate and summarize the published articles that report SWI results for the evaluation of TBI and to determine correlations between clinical status and SWI results. Consequently, our analysis also aimed to identify the appropriate MRI sequences to use in the assessment of patients with TBI. We searched the Medline and Embase online electronic databases for relevant papers published from 2012 onwards. We found that SWI had higher sensitivity than gradient echo MRI in detecting and characterizing microbleeds in TBIs and was able to differentiate diamagnetic calcifications from paramagnetic microhemorrhages. However, it is important that future research not only continues to evaluate the utility of SWI in TBIs but also attempts to overcome the limitations of the studies described in this review, which should help validate the conclusions and recommendations from our analysis.

## INTRODUCTION

Traumatic brain injury (TBI) is defined as any disruption in brain function or other evidence of brain pathology caused by an external physical force. TBI is a major public health issue worldwide, particularly in industrialized countries, because it is one of the leading causes of morbidity and mortality, and there are currently no drugs to treat TBI. These injuries are a ‘male problem’, given that men have a twofold greater risk of sustaining a TBI than women and tend to acquire TBIs at a younger age [1–3].

TBI causes many disorders that add layers of complexity, and it can vary from one patient to another depending on age, sex, severity, and recovery. TBI generally causes widespread diffuse axonal injury, especially in the frontal and temporal lobes, bihemispheric contusions and lesions, cerebral edema, ischemia, cortical spreading depression, spinal cord injury, and hemorrhages in the parenchyma or in the subarachnoid, subdural, or epidural space. A TBI can result in long-term or even life-long physical, cognitive, behavioral, and emotional consequences, including behavioral changes, a lack of initiative, irritability, poor emotional control, and memory impairment. In addition, headache, dizziness, fatigue, sleep disturbances, and balance problems can also occur after TBI. Furthermore, psychological distress, depression, anxiety disorders, and substance abuse are observed, particularly in women <45 and >65 years old. TBIs often co-occur with polytraumatic injuries, including multiorgan injuries, fractures, burns, or amputations, and these injuries require a rehabilitation team specialized in the neurorehabilitation of TBI [4–10]. A few drugs are being tested for TBI, such as methamphetamine, melanocortin, minocycline plus N-acetylcysteine, and cycloserine. These drugs appear to be excellent candidates for clinical trials given their favorable therapeutic windows with multiple outcome measures, which will become a standard component of preclinical drug testing, and enable research on targeted and personalized drugs for neurological diseases and disorders such as TBI [11].

However, differences in the epidemiological data on TBIs are affected by factors such as socioeconomic status, transportation and safety regulations, and the delivery of emergency medical services. The majority of the current statistics on the incidence of TBI originate from industrialized countries; however, a growing body evidence is now emerging from developing countries [12,13]. The primary causes of TBI vary by age, socioeconomic factors, and geographic region, and planned interventions must take into account this variability. The global incidence of TBIs is closely linked to road traffic injuries, falls, and work-related injuries. Road traffic injuries are the most frequently reported cause of TBI; however, there has been a notable increase in the incidence of TBIs caused by falls, from 43% to 54%, particularly in the elderly (>65 years) [14,15]. Evaluation of epidemiological profiles and classification of damage severity and outcomes must be conducted on a frequent basis owing to changes in demographics, approach, and treatments of individuals with TBIs. These assessments can help with the management and choice of preventative tactics for the causes of TBI [16].

Microvascular injury is a near-universal feature of preclinical TBI [17]. TBI can alter the cerebrovascular network, and its acute response to TBI is poorly defined, although emerging evidence suggests cerebrovascular reactivity [18]. Moreover, microvascular ruptures can occur in the subarachnoid space, the brain surface, or in the circulatory system as well as cause redistribution of intraventricular hemorrhages that have penetrated intracranial hematomas, cerebral contusions, and the subarachnoid space. TBIs can be associated with a skull fracture mainly in two or three skull bones, with subdural fluid accumulation that is diagnosed by imaging [19,20].

Magnetic resonance imaging (MRI) has high sensitivity for the detection of epidermal hematoma, subdural hemorrhage, non-hemorrhagic cortical contusions, brainstem injuries, and white-matter axonal injuries (supported by level Ib evidence). MRI is also more sensitive for the detection of hemorrhagic parenchymal contusions (levels Ib–II evidence), all stages of subarachnoid hemorrhage (level II evidence), and subacute subarachnoid hemorrhage (levels Ib–II evidence) [19]. Although TBI results from a primary or other injury, the complicating pathology may be exacerbated by secondary injuries owing to neurochemical and inflammatory processes [21]. Clinically, the severity of TBI can be classified in accordance with the Glasgow Coma Scale (GCS), with mild, moderate, and severe TBIs reflecting GCS scores of 13–15, 9–12, and 3–8, respectively. However, it is increasingly recognized that TBI severity should consider additional factors, including amnesia duration, focal signs, and neuroimaging findings [22].

From a technical perspective, because of its sensitivity to deoxyhemoglobin, hemosiderin, iron, and calcium, susceptibility-weighted imaging (SWI), a term used to describe a susceptibility-weighted MRI technique, is an informative imaging method that is considered superior to conventional MRI for the diagnosis and follow-up of patients with acute, subacute, and prolonged hemorrhage. SWI uses a high-resolution 3D gradient echo sequence with long echo times and flow compensation, combining the magnitude and phase data of MRI to create a phase mask, which has been reported to generate greater sensitivity than that of T2*-weighted gradient echo sequencing [23]. The generated images are also subject to minimum-intensity projection processing, which uses image thicknesses of 3–10 mm with a high signal-to-noise ratio. The protracted echo time in SWI, which is greater than the typical gradient echo sequences at 1.5 Tesla, enhances phase dispersion and shortens protons within heterogeneous fields, enabling the identification of subtle alterations in susceptibility that are seen as signal intensity losses [24]. However, although SWI enhances the accuracy of neuroimaging, it can be limited by the presence of nonspecific magnetic susceptibility sources that can lead to artifacts and impair the interpretation of regions at or surrounding air–tissue interfaces and blooming, resulting in signal cancelation and cloud discrimination of anatomical borders.

The clinical applications of SWI are related to its ability to diagnose and differentiate neurological disorders, including cerebrovascular angioplasties, central nervous system malformations, venous thromboses and infarctions, stroke, and neurodegenerative diseases. It can also identify traumatic microbleeds, even in lesions with particularly high prognostic significance, and the complications of TBI. A TBI results from physical or traumatic injuries to the brain’s surrounding bony structures and associated tissues, which can lead to various sequelae, including simple concussion, acute epidermal hematoma, parenchymal contusions, subarachnoid hemorrhage, diffuse axonal injury, and chronic traumatic encephalopathy [24,25]. The lesion volume detected by SWI correlates closely with injury severity and neurological function, and SWI is able to identify a much higher number of hemorrhagic lesions compared to gradient–echo sequences, which helps optimize prognosis and ongoing management decisions. In contrast to SWI, the sensitivity of MRI to TBI characteristics may temporarily be lower 24–72 h after an injury [26–28].

In view of the limited data available regarding the diagnosis and follow-up of TBI using MRI, the aims of this systematic review were to evaluate and summarize the published articles that report SWI results for the evaluation of TBI and to determine correlations between clinical status and SWI results. Consequently, our analysis also aimed to identify the appropriate MRI sequences to use in the assessment of patients with TBI.

## MATERIAL AND METHODS

### Search strategy

**Figure 1:**
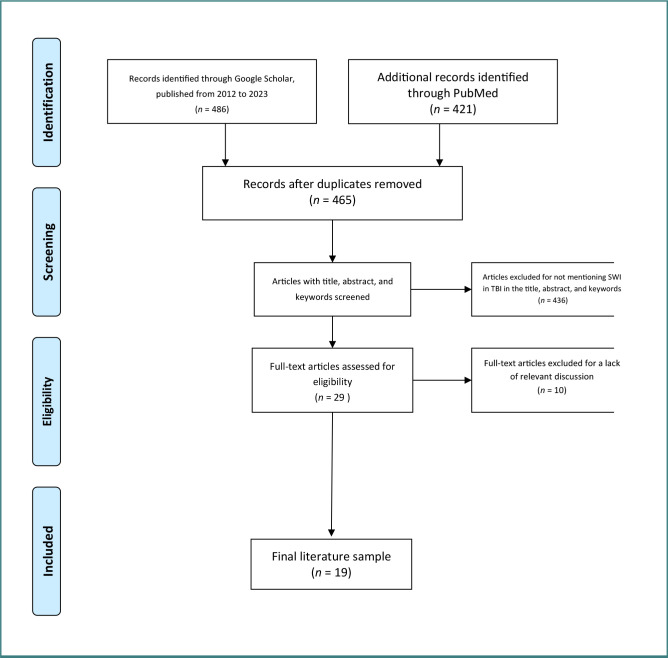
Flowchart outlining the protocol adopted in this systematic review based on the PRISMA guidelines

In this systematic review, the online Google Scholar and PubMed electronic databases were searched using the terms 'susceptibility-weighted imaging' OR 'SWI' AND 'traumatic brain injury' OR 'TBI' OR 'traumatic head injury' OR 'traumatic intracranial hemorrhage' and others (Figure 1). Syntax and Boolean operators were used because they are known to generate accurate and precise results [29].

The inclusion criteria of the study were articles published in and after 2012; written in English; with primary research information, including data regarding individuals with TBI; free full-text; and those published in what we considered to be scholarly journals. Young and adult populations were included, as TBI with worsened conditions is usually observed in these groups. These individuals require effective MRI scanning to establish an accurate diagnosis and choose a suitable treatment. The exclusion criteria were studies not written in English, those published before 2012, and those that contain secondary information, such as systematic reviews and meta-analyses, animal studies, conference abstracts, magazines, books, encyclopedias, and non-academic articles.

Articles published before 2012 were excluded because they contained backdated information, which may lead to errors in the presentation of findings. Non-academic articles were excluded because they contained descriptive information and included only scientifically-approved data via presentations of enhanced scientific evidence.

### Study selection

Each article was independently evaluated by at least two reviewers, based on the title, abstract, and full text. Articles were considered for inclusion based on the Preferred Reporting Items for Systematic Reviews and Meta-Analyses (PRISMA) reporting guidelines for systematic reviews, focusing on patients with TBI or those who had undergone SWI. Disagreements between reviewers were resolved by consensus or by the decision of a third independent reviewer.

### Data extraction

Study details, such as location, duration, design, sample size, data sources, TBI, imaging methods (MRI or SWI), epidemiological data (prevalence, mortality, demographic information, and mechanism of injury), and TBI-related outcomes (detection of hemorrhagic lesions, visualization of veins, and cerebral traumatic microbleeds) were extracted from the included studies as reported. One reviewer performed the data extraction, which was then reviewed by the second reviewer.

### Quality assessment

The Critical Appraisal Skills Program checklist was used to assess all articles meeting the inclusion criteria for bias. One reviewer independently assessed the quality of all included studies, which were then reviewed by the second reviewer. The possibility of bias associated with each article is acknowledged in the narrative synthesis and discussion of this review, and no articles were disqualified from the review or the narrative analysis because of their potential for bias.

## LITERATURE REVIEW

### SWI is beneficial for TBI diagnosis

Most evidence supporting the utility of SWI was published >10 years ago; hence, SWI should already be a part of routine radiological practices. The following literature review discusses older studies and concludes with the most recent studies.

For diffuse axonal injury, the torsional forces exerted upon cerebral axons via rapid acceleration and deceleration of the cranium can lead to microhemorrhages within certain areas, such as the cerebral gray-white-matter junction, corpus callosum, basal ganglia, and dorsolateral brainstem, which are detected with greater sensitivity by SWI than by computed tomography (CT) and gradient echo MRI sequences [30].

In a previous earlier study, Tong *et al*. (2003) compared the accuracy of SWI versus conventional gradient echo MRI in characterizing posttraumatic diffuse axonal injury in a small cohort of seven children. The subjects had a mean GCS of 7 and were evaluated at a mean of 5 days post injury. The results showed that a much higher number of hemorrhagic lesions were found by SWI than by gradient echo MRI, with SWI identifying 1,038 lesions with a blood volume of 57,946 mm^3^ and gradient echo MRI identifying only 162 lesions, representing half of the apparent blood volume (28,893 mm^3^). Statistical analysis revealed that the difference in hemorrhagic lesion detection and related blood volume significantly favored SWI (*P* = 0.004 and *P* = 0.014, respectively). The difference between the sequences was largely attributed to the superior sensitivity of SWI in detecting small hemorrhagic lesions (Figure 1), almost 60% of lesions identified by SWI being <10 mm^2^, whereas a similar proportion of lesions detected using gradient echo MRI were 10–20 mm^2^ in size [28]. In a study involving 79 patients with mild-to-severe TBI, SWI identified TBI-related lesions in almost one third of the patients in whom fluid-attenuation inversion recovery (FLAIR) MRI was unable to detect any lesions. Greater overall and frontal SWI volumes were related to the severity of TBI [31]. In another study, researchers evaluated the utility of SWI in a cohort of active military personnel who had suffered mild TBIs (mTBIs) during duty and found that SWI was markedly superior to FLAIR MRI in highlighting cerebral abnormalities comprising white matter hyperintensities [26]. In another study involving 147 subjects from the military who had sustained TBIs, SWI combined with a multi-scale vessel enhancement filter resulted in significantly better discrimination of venous volumes across all brain regions in the patients than in the controls, which effectively helped characterize chronic pathological changes related to iron deposition and astroglial scarring [32].

Another study, which performed a blinded comparison of SWI and gradient echo MRI in 40 children and adolescents with diffuse axonal injuries, found that SWI detected a significantly higher number of hemorrhagic lesions and, notably, image interpretation was not impaired by the enhanced visualization of veins, consistent with the original purpose of SWI. Moreover, the authors found that SWI was less susceptible to blooming artifacts than gradient echo MRI, although it was limited by a more protracted image acquisition time [33]. Advanced neuroimaging showed that the correlation of traumatic microbleed features in patients with TBI was higher with SWI than with gradient echo MRI because SWI is able to better differentiate between hemorrhage-related blood products and the surrounding brain parenchyma. SWI can identify traumatic microbleeds even in lesions with a particularly high prognostic significance [25]. Similarly, SWI has been shown to be markedly useful for assessing children with TBIs and to have a sixfold greater sensitivity for detecting microhemorrhages than T2*-weighted gradient echo MRI [3]. Studies have also shown that injury severity and neuropsychological outcomes at 6 and 12 months in individuals with TBI correlate better with SWI than with gradient echo MRI findings, and that the number of hemorrhagic lesions detected was strongly associated with the GCS and cognitive, physical, and psychological function at discharge [25].

Another study found that in patients with TBI, SWI was notably useful for detecting pathology within the brainstem, which could not be identified by other MR sequences. This is an important benefit given that brainstem involvement can be associated with poorer outcomes [34]. Moreover, the authors showed that in some cases, SWI was also able to detect hemorrhagic lesions in the lateral ventricles and the subarachnoid space that were not identified by CT, and these findings influenced clinical decision-making and management for several patients [34]. However, it is important to consider that these studies are limited by their small sample sizes, which could invalidate the results owing to type II errors. Another study found that SWI outperformed CT scans in detecting intracranial hemorrhage in patients with TBI, confirming that SWI is extremely informative owing to its high sensitivity to deoxyhemoglobin, hemosiderin, iron, and calcium, making it superior in the diagnosis and follow-up of patients with acute, subacute, and chronic hemorrhage. A larger amount of neurological information and details for detecting subarachnoid hemorrhage, epidermal hematoma, subdural hemorrhage, and cerebral contusions were obtained by SWI than by CT, therefore performing SWI is recommended before control CT in TBI cases [35].

### SWI and mTBI

The detection of microhemorrhages by SWI for other forms of TBI has also proven to be clinically important, for example in mTBI, which can often present with a latency phase and a later neurophysiological demise as a result of delayed and compensated clinical signs. For example, Hageman *et al*. found that conventional MRI could not identify several hemorrhages in a motorcyclist with traumatic head injury using gradient-echo MRI, revealing only one small bleed. By contrast, SWI was able to highlight several injurious veins close to large venous septa and in the periphery of the cerebral parenchyma, which was attributed to the shearing forces of the injury acquired [27]. In addition, a 2012 study found that SWI had a six times greater ability to detect hemorrhagic diffuse axonal injuries than other MRI techniques, highlighting its advantage in identifying microhemorrhages that are not discernible with other MRI techniques, as well as the subtle nature of mTBI abnormalities [36].

In another study, the authors explored the utility of SWI and diffusion tensor imaging in several patients with a history of mTBIs. They found that SWI was able to highlight the small foci of hemosiderin deposits, which comprised evidence of a previous microbleed [27]. However, this study was markedly limited by its design, as SWI was evaluated only among a few patients with head injuries. Similarly, in 2021, Virani *et al*. evaluated the utility of SWI in 106 children with TBIs of various severities. They reported that lesions were detected in 35% of cases, comprising 19% of patients with mild injury, 46% with mild but complex injury, 59% with moderate injury, and 54% with severe injury, suggesting that sensitivity was high across all severities of brain injury but favored detection in patients with more severe injuries [37]. This view was reflected in the mean number of lesions detected, which ranged between 0.8 and 3.2 for patients with mild brain injury, compared with 8.9 and 14 for patients with moderate and severe injuries, respectively. A similar trend was observed for the volume of lesions identified, with a mean lesion volume of 49.3–218.1 mm^3^ for patients with mild injury compared with 658 mm^3^ and 2,653 mm^3^ for patients with moderate and severe injuries, respectively. Nevertheless, SWI has limitations in detecting microvascular changes and microhemorrhages, such as a relatively long acquisition time and high sensitivity to motion artifacts, which vary depending on factors such as the patient’s condition and the detection capabilities of available sequences [38].

### SWI, GCS, and microbleeds

In 2021, Virani *et al*. reported that the number and volume of lesions detected by SWI significantly correlated with GCS, need for surgical intervention, length of inpatient stay, and duration of mechanical ventilation. However, no such associations were observed for age at injury onset, neurological signs or symptoms, and loss of consciousness at the initial presentation [37]. Furthermore, the findings on SWI, in combination with GCS, accounted for 7% of the variance in the Wechsler Abbreviated Intelligence Scale, which was statistically significant (*P* = 0.04). However, the validity of this study may have been adversely affected by the differences in post-injury imaging times between the subjects, which could have affected the findings on SWI.

Few studies have reported the utility of SWI in adults with mTBIs, and some of them had mixed results. In one such study, Salehi *et al*. found that diffusion tensor imaging was able to identify microhemorrhagic changes among some of the 14 included subjects with mild brain injuries. Still, SWI was unable to detect any of the abnormalities [38]. Despite this, two other studies showed that SWI was able to identify microbleeds in boxers who sustained repetitive mTBIs in the form of concussions, which could not be detected by fusing T2 fast-spin-echo or gradient echo sequences [39,40]. Similarly, a study involving a cohort of ice hockey players who had been involved in marked physical contact that had resulted in mild traumatic head injuries showed that SWI was able to identify multiple cerebral microbleeds with clear enhancement on acquired images, although no comparison with other sequences or imaging modalities was performed [41].

Finally, several more recent studies revealed similar superiority of SWI in detecting microhemorrhages in patients with mild and severe forms of TBIs and correlated these aberrations with prognostic outcomes, highlighting that the choice of MRI sequence remains a contemporary topic and issue in radiological practice [32,42–45]. The first of these studies recruited a cohort of 25 patients with hemorrhagic diffuse axonal injury who exhibited unclear changes upon initial CT scanning but had hemorrhagic foci on MRI, and it was observed that the use of SWI detected the highest number of microbleeds among all MR sequences, including gradient echo, diffusion-weighted, FLAIR, and conventional T2W and T1W MRI. However, SWI did not detect a significantly greater number of hemorrhagic lesions within the brain stem or cerebellar regions than other MRI sequences (*P* > 0.05).

### Pediatric TBIs

A prospective study that evaluated the association between clinical and imaging findings among a cohort of 105 children with TBIs diagnosed by SWI found that the severity, number, and volume of microhemorrhagic lesions was significantly correlated with executive function at 6 months post injury [44]. In the last study that we included in this review, the authors aimed to reduce the rate of false positives when diagnosing cerebral microbleeds by integrating SWI into the diagnostic protocol [45]. The results showed that SWI had high sensitivity for detecting and characterizing microbleeds because it was able to differentiate diamagnetic calcifications from paramagnetic microhemorrhages. The protocol was evaluated among 41 patients with brain injuries, and the best model had a sensitivity of 96% and a precision of 71%, lowering the false-positive rate to 1.6 per case.

SWI has also been found to reliably predict longer-term outcomes in children with non-accidental TBIs. In a study involving 101 children, the authors found that SWI detected cerebral microhemorrhages in 29%, extradural bleeds in 65%, retinal hemorrhages in 51%, and ischemic injuries in 35% of non-accidental injury cases [46]. Logistic regression analyses revealed that the presence of microhemorrhages detected by SWI was the most significant predictor of neurological outcomes at 6 months, with an overall accuracy of 92.5%. However, the study was affected by selection bias owing to the inclusion of patients with more severe forms of brain injury, as well as bias related to small sample size and confounders owing to the possibility of previous undocumented head injuries.

Given that most of the recently published studies were affected by suboptimal sample sizes and retrospective cross-sectional designs, it is recommended for future research to prioritize systematic reviews and meta-analyses. However, implementing SWI into routine head trauma protocols is only feasible for prognostic purposes in the case of patients who were already examined using CT, because CT is more readily available and has markedly shorter acquisition times compared to MRI [47]. Therefore, CT should remain the first-line imaging modality for patients with acute head trauma, as recommended by current guidelines. However, the guidelines should be adapted to incorporate SWI as the first-line MRI protocol for prognostic purposes, given the limitations of CT in describing and evaluating the severity of certain head injuries, such as small hemorrhagic contusions, and the fact that MRI has improved diagnostic sensitivity for non-hemorrhagic contusions and shear-strain injuries. CT and MRI can be used to predict clinical outcomes, and there is particular interest in advanced applications of both techniques that may greatly improve the sensitivity of conventional CT and MRI for the diagnosis and prognosis of TBI [48,49] (Table 1).

**Table 1:** The sensitivity of conventional CT and MRI for the diagnosis and prognosis of TBI

Study	Detection of hemorrhagic lesions (n/%)		Sensitivity for detecting hemorrhagic lesions (qualitative)	
	SWI	Other MR sequences	SWI	Other MR sequences
**Tong *et al*. (2003)^28^**	1,038	162 (GE)	Superior	Inferior (GE)
**Ramasamy *et al*. (2018)^33^**	–	–	Superior	Inferior (GE)
**Kirov *et al*. (2018)^50^**	–	–	Superior: sixfold	Inferior (GE and T2)
**Barnes and Haacke (2009)^51^**			Superior	Inferior (GE)
**Hageman *et al*. (2022)^27^**			Superior	Inferior (DT)
**Virani *et al*. (2021)^37^**	19% mild TBI, 59% moderate TBI, 54% severe TBI	No comparator	High	N/A
**Bartnik–Olson *et al*. (2021)^46^**	29%	No comparator	High	N/A
**Salehi *et al*. (2016)^38^**	0	14	Inferior	Superior (DT)
**Koerte *et al*. (2016)^40^**	–	–	Superior	Inferior (T2 FSE and GE)
**Kung *et al*. (2018)^41^**	–	–	Superior	Inferior (T2 FSE and GE)
**Tao *et al*. (2015)^42^**	632	210 (GE) 171 (DW) 141 (FLAIR) 111 (T2) 86 (T1)	Superior	Inferior (all comparators)
**Tate *et al*. (2017)^43^**	–	–	Superior	Inferior (FLAIR)
**Liu *et al*. (2019)^45^**	–	–	High	No comparator
**Resch *et al*. (2019)^44^**	–	–	High	No comparator
**Liu *et al*. (2019)^32^**	–	–	High	No comparator

DT, diffusion tensor; GE, gradient echo; T2 FSE, fast spin-echo T2-weighted Dixon imaging

## DISCUSSION

The primary objectives of this systematic review were to assess and synthesize the extant literature pertaining to SWI findings in the context of TBI and to ascertain potential correlations between clinical indicators and SWI outcomes. Additionally, our analysis aimed to delineate the optimal MRI sequences for the comprehensive evaluation of individuals afflicted with TBI. The review yielded important findings, emphasizing the utility of SWI in the diagnosis of pathologies within the brainstem, an achievement unattained by conventional MRI sequences. Furthermore, SWI demonstrated a unique capability to discern minute hemosiderin deposits that serve as markers of previous microbleeds. In comparison to other MRI sequences, SWI exhibited superior sensitivity in the identification of microbleeds, a salient aspect of TBI evaluation. Nevertheless, our analysis did not reveal a statistically significant difference in the detection of hemorrhagic lesions within the brainstem or cerebellar regions compared to alternative MRI sequences (*P* > 0.05). In addition, SWI has potential in the detection of diamagnetic calcifications in pediatric TBI cases, with results affirming its increased sensitivity and specificity in discriminating diamagnetic calcifications from paramagnetic microhemorrhages, thereby augmenting diagnostic accuracy.

The limitations of this review include the risk of several forms of bias, given that non-English language studies were excluded, and integrated care in areas where English is not a primary language may have been missed, thus limiting the comprehensiveness of the review. Data from the body of literature are sparse and often difficult to translate into clinical practice because of the retrospective designs of the studies and the small number of enrolled patients.

## CONCLUSION

In summary, this systematic analysis critically evaluated the evidence regarding the efficacy of SWI in TBIs. We concluded that this MRI sequence should be adopted in routine imaging protocols. However, it is important that future research not only continues to evaluate the utility of SWI in TBIs but also attempts to overcome the limitations of the studies described in this review, which should help validate the conclusions and recommendations from our analysis.
